# A computerized tool for the systematic visual quality assessment of infant multiple-breath washout measurements

**DOI:** 10.3389/fped.2024.1393291

**Published:** 2024-06-06

**Authors:** Marc-Alexander Oestreich, Isabelle Doswald, Yasmin Salem, Noëmi Künstle, Florian Wyler, Bettina S. Frauchiger, Anne-Christianne Kentgens, Philipp Latzin, Sophie Yammine

**Affiliations:** ^1^ Division of Paediatric Respiratory Medicine and Allergology, Department of Paediatrics, Inselspital, Bern University Hospital, University of Bern, Bern, Switzerland; ^2^ University Children’s Hospital Basel (UKBB), University of Basel, Basel, Switzerland

**Keywords:** pulmonary function testing, infant lung function, SF_6_-MBW, lung clearance index, functional residual capacity, quality control, sigh, breathing pattern

## Abstract

**Background:**

Multiple-breath washout (MBW) is a sensitive method for assessing lung volumes and ventilation inhomogeneity in infants, but remains prone to artefacts (e.g., sighs). There is a lack of tools for systematic retrospective analysis of existing datasets, and unlike N_2_-MBW in older children, there are few specific quality control (QC) criteria for artefacts in infant SF_6_-MBW.

**Aim:**

We aimed to develop a computer-based tool for systematic evaluation of visual QC criteria of SF_6_-MBW measurements and to investigate interrater agreement and effects on MBW outcomes among three independent examiners.

**Methods:**

We developed a software package for visualization of raw Spiroware (Eco Medics AG, Switzerland) and signal processed WBreath (ndd Medizintechnik AG, Switzerland) SF_6_-MBW signal traces. Interrater agreement among three independent examiners (two experienced, one novice) who systematically reviewed 400 MBW trials for visual artefacts and the decision to accept/reject the washin and washout were assessed.

**Results:**

Our tool visualizes MBW signals and provides the user with (i) display options (e.g., zoom), (ii) options for a systematic QC assessment [e.g., decision to accept or reject, identification of artefacts (leak, sigh, irregular breathing pattern, breath hold), and comments], and (iii) additional information (e.g., automatic identification of sighs). Reviewer agreement was good using pre-defined QC criteria (κ 0.637–0.725). Differences in the decision to accept/reject had no substantial effect on MBW outcomes.

**Conclusion:**

Our visual quality control tool supports a systematic retrospective analysis of existing data sets. Based on predefined QC criteria, even inexperienced users can achieve comparable MBW results.

## 1 Introduction

Multiple-breath washout (MBW) is an established test to evaluate the functional residual capacity (FRC) and ventilation distribution of the lungs by assessing the washout of an inert tracer gas (1, 2). Its main outcome, the lung clearance index (LCI), is a sensitive marker of early structural lung disease (1, 3) which is used for clinical surveillance of patients with cystic fibrosis (CF) (4, 5) as well as an outcome in clinical trials of new therapies (6–8). Unlike other lung function tests such as conventional spirometry, MBW requires only passive cooperation and relaxed tidal breathing and is feasible from infancy on (2, 3, 5, 9–11). However, it can be time consuming and challenging to obtain measurements of adequate quality in infants (3, 12).

While most infants have regular breathing patterns during mandatory non-REM sleep (13), artefacts such as sighs, breath holds, or leaks might occur and affect MBW results (Supplementary Figure S1) (1, 8). Current ATS/ERS consensus guidelines recommend the exclusion of measurements containing any evidence of artefacts within a critical test phase. Sulfur hexafluoride (SF_6_)-based MBW is currently the preferred washout method in infants. But unlike for nitrogen MBW in older children and adults, few specific definitions of visual quality criteria for artefacts exist in infants (1–3, 10). While numerically based quality criteria (e.g., end-of-test or FRC variability) are well-defined and therefore easy to verify for the operator (1), decision making on subjective visual artefacts (e.g., leaks or irregular breathing) remains difficult and may depend on experience.

Currently, there are two software packages available for the analysis of infant SF_6_-MBW measurements: The historic WBreath (ndd Medizintechnik AG, Zurich, Switzerland) and the more recent Spiroware software (Eco Medics AG, Duernten, Switzerland), both applying indirect measurement principles for the inert tracer gas. Due to fundamental differences in the signals used and algorithms applied, the data of the two systems cannot be used interchangeably (14, 15). Moreover, both software packages have recently received relevant updates affecting the main outcomes, so that previously collected data needs to be reanalyzed (14, 16). Neither application provides an option for systematic and detailed visual quality assessment beyond a global inclusion/exclusion criterion (in Spiroware software) (17), leaving the visual quality control assessment and documentation to the operator.

We therefore aimed to develop a computerized tool for the systematic assessment of predefined visual quality control (QC) criteria of infant SF_6_-MBW measurements, to evaluate agreement in three independent users performing visual quality assessment of infant SF_6_-MBW collected in both available setups (Spiroware and WBreath), and to assess whether different visual QC assessment has an effect on MBW outcomes.

## 2 Methods

### 2.1 Study population

This was a retrospective, observational study of infant SF_6_-MBW data from previously described cohorts of healthy infants (Basel-Bern Infant Lung Development (BILD) cohort (18, 19) and infants diagnosed with CF (Swiss Cystic Fibrosis Infant Lung Development (SCILD) cohort (20); Supplementary Table S1). The reference examiner (MO) selected part of the dataset to ensure a minimum number of measurements with artefacts present, while the additional measurements were randomly selected to obtain a total of 200 SF_6_-MBW trials per setup (Spiroware and WBreath; Table 1). Each dataset included measurements from study visits at six weeks of age (BILD cohort) and six weeks or one year of age (SCILD cohort). The Ethics Committee of the Canton of Bern, Switzerland approved the study protocol (B201901072, PB_2017-02139) and parents gave written consent.

**Table 1:** Characteristics of the reference dataset.

	Spiroware setup (*n* = 200)		WBreath setup (*n* = 200)	
	Washin	Washout	Washin	Washout
Unacceptable trials	*54* *(27%)*	*74* *(37%)*	*41* *(20.5%)*	*75* *(37.5%)*
Leak	22 (11%)	27 (13.5%)	14 (7%)	33 (16.5%)
Sigh	17 (8.5%)	28 (14%)	18 (9%)	20 (10%)
Irregular breathing	10 (5%)	13 (6.5%)	3 (1.5%)	7 (3.5%)
Breathhold/Apnoea	1 (0.5%)	2 (1%)	1 (0.5%)	10 (5%)
Incomplete trial	4 (2%)	4 (2%)	4 (2%)	4 (2%)
Error	–	–	1 (0.5%)	1 (0.5%)
Acceptable trials	146 (73%)	126 (63%)	159 (79.5%)	125 (62.5%)
Artefact outside critical test phase	6 (3%)	6 (3%)	2 (1%)	4 (2%)
No artefact	140 (70%)	120 (60%)	157 (78.5%)	121 (60.5%)

Data are presented as *n* (%total), unless otherwise stated. Per measurement occasion, one trial was included for analysis, in total *n* = 200 per setup (Spiroware and WBreath). Excluded trials may feature more than a single artefact in the critical phase of the washin or washout.

### 2.2 MBW measurements

MBW measurements were performed during natural, non-REM sleep at six weeks of age and under sedation with chloral hydrate (75 mg/kg; per rectum) at one year of age in accordance with current ATS/ERS standards (1). While sleeping in supine position with head in midline, infants breathed through a facemask (open cuff silicone mask, size 1; Draeger AG, Luebeck, Germany). Flow and molar mass (MM) signals were measured by an ultrasonic flowmeter [Exhalyzer D, Eco Medics AG; with either WBreath 3.28.0 (ndd Medizintechnik AG) or Spiroware 3.2.1 software (Eco Medics AG)] using 4% SF_6_. The operator aimed for 2–3 valid trials per subject and setup.

### 2.3 Data analysis and display options for assessment

**Figure 1:**
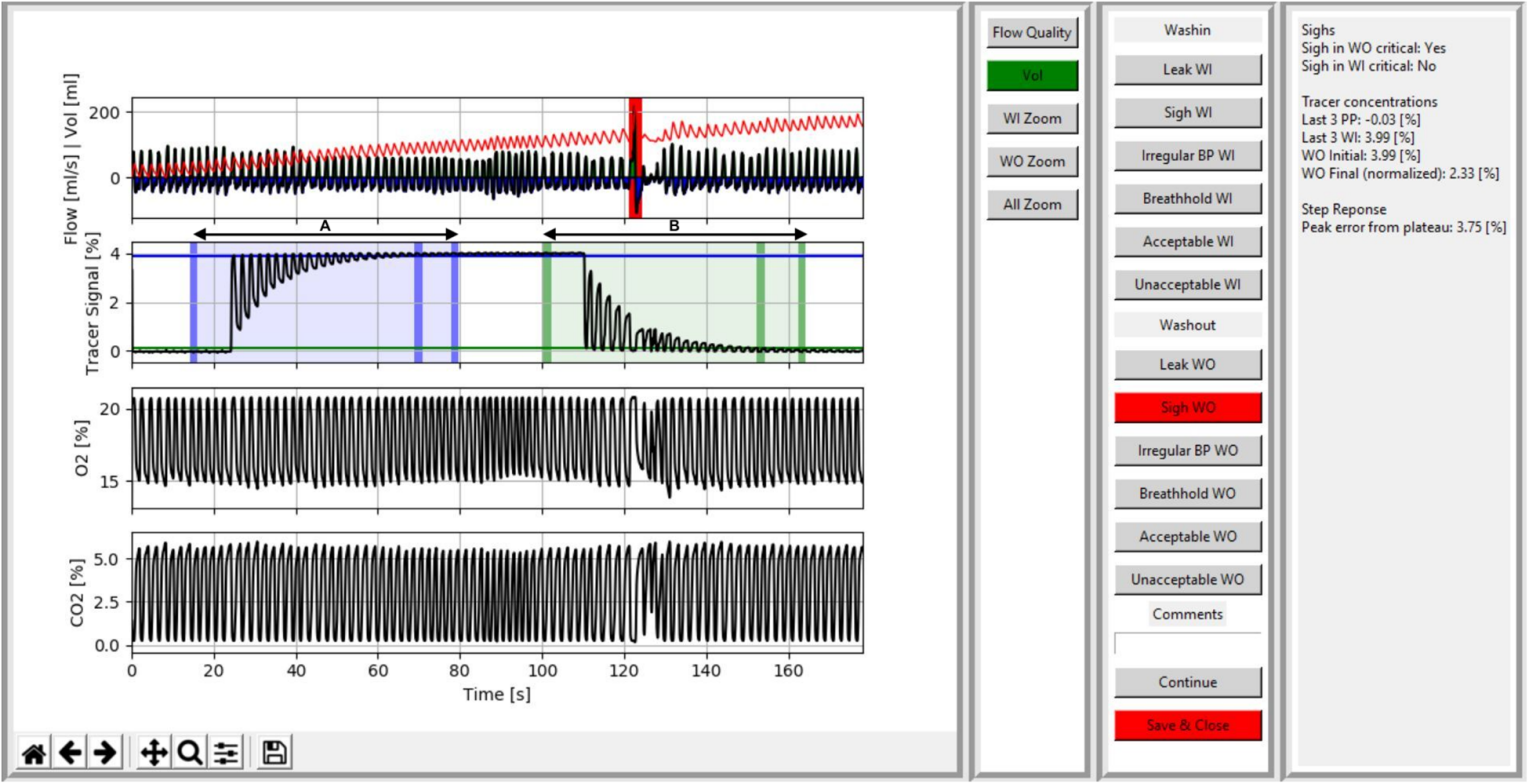
Screenshot of the visual quality control tool visualizing signal processed data of a spiroware infant SF_6_-MBW trial with a sigh in the critical phase of the washout. Data of a trial including the washin (**A**) and washout (**B**) phase is shown. In the first panel, signal traces for flow (ml/s) and volume (ml), a computed tracer gas concentration (%), oxygen (%), and carbon dioxide (%) are shown. The critical periods are highlighted by blue (**A**) and green (**B**) areas in the tracer signal, with vertical bars indicating (i) the beginning of the critical phase, (ii) the 2.5% criterion, and (iii) the end of the critical phase. A sigh (= a breath with a significant increase in tidal volume above 1.5 times the median tidal volume) during the washout is marked in red by the automatic sigh detection and the user is provided with a comment (box on the right). The second and third panels provide the user with display options (e.g., zoom and a heat map of tidal volume per breath), systematic assessment options (e.g., to accept/reject, identify artefacts (leak, sigh, irregular breathing pattern, breath hold), and comment, and additional information for the operator (e.g., automatic identification of sighs, deviations in step response-correction, or molar mass steps between phases).

Per measurement occasion, one trial was included in the study dataset for analysis. Calculation of outcome parameters, LCI and FRC, were performed in the software versions 3.52.3 for WBreath (14), and 3.3.1 for Spiroware (16, 21). For visual quality control, we developed custom Python scripts to display data gathered by both setups by reading the exported text files of each trial. Raw data containing text files in Spiroware (A-files) consisted of unprocessed flow, molar mass (MM), oxygen (O_2_), and carbon dioxide (CO_2_) signals (13). In WBreath, raw data consisted of corrected flow and MM signals (14) (ASCII export) after applying signal processing which includes BTPS-correction, temperature simulation, and a step-response correction according to standard protocols (14). Besides the flow and volume trace, we additionally displayed the following signals in Python: for Spiroware files tracer gas, O_2_, and CO_2_ concentrations; for WBreath mainstream MM and a computed tracer gas concentration signal. We programmed an automated identification of the critical test phases for both washin [Figure 1, blue area (A)] and washout [Figure 1, green area (B)] defined as five breaths before washin/washout start to five breaths after reaching the test-end criterion [i.e., 1/40th of the starting tracer gas-concentration)]. Further, we developed a heat map visualization based on the tidal volume difference of each breath to the median tidal volume over the measurement. Within the flow signal trace, we developed the option of automated sigh detection, and observer-based identification of irregular breathing and breath hold assisted by visual quality criteria.

### 2.4 Systematic assessment of MBW trials

Based on current ATS/ERS consensus recommendations (1), we developed a step-wise workflow for the systematic assessment of visual quality criteria (Supplementary Summary). All SF_6_-MBW trials were preliminarily evaluated, categorized, and annotated by the reference examiner (MO; Table 1). The washin and washout phase within each trial were assessed separately. Excluded trials may feature more than a single artefact in the critical phase of the washin or washout, whereby the examiners were instructed to identify all detected artefacts. For an acceptable trial and the calculation of outcomes, all qualitative criteria had to be met within the critical test phase of the washout. Outside the critical test phase, the following qualitative criteria could deviate and, depending on the individual case, the washout still be classified as acceptable: sigh, irregular tidal breathing, and breath hold. An error was defined as empty or erroneous signal trace(s) and led to exclusion of the trial.

### 2.5 Statistical analysis

Three blinded reviewers (MBW experienced: YS, ID; MBW novice: NK) evaluated the same set of 400 MBW trials using the tool and following the quality check-list (Supplementary Summary). For each trial, the reviewers assessed the washin and washout separately and documented their (i) decision to accept/reject, (ii) identified visual artefacts, and (iii) reason for exclusion using the developed tool. In a next step, we compared the interrater agreement for the decision to accept/reject between the reference (MO) and each of the three reviewer (YS, ID, NK) using Cohens Kappa (κ) and between all reviewers using Brennan and Predigers Kappa coefficient (22). We interpreted the Kappa coefficient between 0.41 and 0.60 as moderate, 0.61 and 0.80 as substantial, and 0.81 and 1.0 as almost perfect agreement. Interrater agreement was then compared between (a) the setups (WBreath and Spiroware), and (b) between healthy infants and infants with CF. The reported percent agreement is the percentage of all identically rated decisions (to accept or to reject the washin or the washout) among the reference and the three reviewers. Finally, we examined whether the systematic quality control had an influence on LCI and FRC as main outcome parameters of MBW. For this, we compared mean LCI/FRC of all acceptable washouts between the reviewers using paired *t*-tests per setup, and per disease group. Additionally, the reference examiner (MO) investigated the total time required to (i) boot the analysis software (WBreath, Spiroware, and the developed visual QC tool) and import raw data, and (ii) perform visual quality control and document (a) the decision to accept/reject, (b) identified visual artefacts, and (c) the reason for exclusion in a subset of five files per setup (randomly chosen from the main dataset). The total time required to perform a visual quality control and document the findings and decision to accept/reject was compared by paired *t*-tests (Supplementary Table S2). Statistical analysis was performed using STATA 16 (StataCorp, College Station, USA) and GraphPad Prism 9 (GraphPad Software, San Diego, USA). A *p* ≤ 0.05 was considered significant.

## 3 Results

### 3.1 Visual quality control tool

The assessment of infant SF_6_-MBW measurements can be divided into (i) the analysis of gathered raw data (signal processing and outcome calculation), (ii) a numeric QC based on pre-defined thresholds (e.g., equilibrium of exogenous washin gas, or reaching a tracer gas concentration below 2.5% at the end of the washout), and (iii) a visual QC for artefacts (e.g., leaks, sighs, irregular breathing patterns, or breath holds; Table 2). We developed a software package for the visualization of (raw Spiroware and signal processed WBreath) signal traces supporting a systematic assessment of pre-defined visual QC criteria, which is now available to researchers (https://doi.org/10.6084/m9.figshare.22193737.v2). A detailed summary on applied criteria is provided in the online Supplementary.

**Table 2:** Visual quality criteria for the systematic evaluation of SF_6_-MBW trials.

	Criteria for test acceptability	
	ATS/ERS consensus statement (1)	Adapted criteria for visual quality control assessment
General	• Regular breathing pattern. • Stable tidal volume and end-expiratory lung volume. • Equilibration of exogenous washin gas within the breath cycle with a variability <0.04% relative to the mean inspired concentration. • No evidence of significant trapped gas release with larger breaths. • No coughing. • Sufficient interval between trials to allow inert gas concentration to return to baseline values.	
Critical phase	• Ten breaths prior to achieving equilibration and the first ten breaths of the washout.	• Five breaths before washout start to five breaths after reaching the test-end criterion (1/40th of the starting tracer gas-concentration) of the washout.
Leak	• No evidence of leak or excessive drift during the test. A leak may be indicated by: Failure of equilibration between inspiratory and expiratory inert gas concentrations, a sudden drop in inspiratory inert gas concentration during the washin, or an increase in deadspace volume during the washout.	• No evidence of leak during the test. • Leak was defined as a step change or irregular slope of the volume trace with no other artefacts present.
Sigh	• No evidence of sighs during critical periods of the washin/washout.	• No evidence of sighs during the critical periods of the washin/washout. • A sigh was defined as a marked increase in tidal volume (at least 1.5-fold of the median tidal volume) with no other artefacts present.
Irregular breathing	• Regular breathing pattern. • Stable tidal volume. • No irregular small volume breath immediately prior to starting the washout.	• Regular breathing pattern. • Stable tidal volume. • No irregular small volume breath immediately prior to starting the washin or washout. • Irregular breathing was defined as irregularities in the flow signal that affect other signals with no other artefacts present.
Breath hold/apnoea	• No evidence of apnoeas during critical periods of the washin/washout.	• No evidence of breath holds or apnoeas during critical periods of the washin/washout. • A breath hold was defined as flattening/pause of the flow signal for the duration of at least two regular tidal breaths that affects other signals.

Summary and definitions of applied quality control criteria for visual artefacts in infant SF_6_-MBW measurements as proposed by the current ATS/ERS consensus statement for inert gas washout (https://doi.org/10.1183/09031936.00069712) and our adapted criteria.

The user interface features four sections: (i) signal traces of flow, volume, and tracer gas over time, as well as MM (for WBreath data) and O_2_ and CO_2_ (for Spiroware data), (ii) display options (e.g., zoom and a heat map visualization of tidal volume per breath), (iii) systematic assessment options (for both washin and washout) to accept/reject, identify artefacts (leak, sigh, irregular breathing pattern, breath hold), and comment, and (iv) additional information for the operator [e.g., automatic identification of sighs, deviations in step response-correction, or MM steps between phases (Figure 1)]. A color-coded critical phase for washin (blue) and washout (green), as well as the achievement of the 2.5% criterion (1), provide the operator with visual support (automatic phase detection; Figure 1).

### 3.2 Study population

The characteristics of the study population for both setups are summarized in Supplementary Table S1. While part of the dataset were selected by the reference examiner (to ensure a minimum number of measurements with artefacts present), the additional measurements were randomly selected to obtain 200 SF_6_-MBW trials per setup (Table 1). For WBreath, the study population included more healthy children than patients with cystic fibrosis, while for Spiroware the groups were comparable. On average, the patients with cystic fibrosis were older than the healthy controls.

### 3.3 Systematic evaluation of MBW trials

The reference dataset included a comparable amount of acceptable and unacceptable trials in both setups (Table 1). The most common artefacts leading to trial exclusion in both setups were leak and sigh (Table 1). Using the newly programmed tool for the systematic visual quality assessment by the three independent reviewers, the interrater agreement ranged from 81.1% to 86.3% among the reviewers (kappa Spiroware washout: 0.637; kappa WBreath washout: 0.653; Table 3). The interrater agreement for the decision to accept/reject the washout between all individual reviewers was similar in WBreath (82.7%) compared to Spiroware (81.8%). Comparison of the interrater agreement (decision to accept/reject the washout) between healthy children and children with CF showed no difference within the setups (WBreath *p* = 0.055, Spiroware *p* = 0.261; Table 3). For both infant MBW setups, the time required to perform visual quality control and documentation of findings (e.g., the decision to accept/reject) was substantially shorter when using the newly developed tool compared to the standard analysis software WBreath or Spiroware (paired *t*-test, *n* = 5, WBreath *p* < 0.001; Spiroware *p* = 0.031; Supplementary Table S2).

**Table 3:** Interrater agreement after independent review.

	Spiroware setup (*n* = 200)				WBreath setup (*n* = 200)			
	Washin		Washout		Washin		Washout	
Reviewer	% (95% CI)	*Κ* (95% CI)	% (95% CI)	*Κ* (95% CI)	% (95% CI)	*Κ* (95% CI)	% (95% CI)	*Κ* (95% CI)
1 (exp) vs. Ref	85.0	0.57	82.5	0.62	89.5	0.67	81.5	0.61
2 (exp) vs. Ref	90.5	0.73	88.5	0.74	89.0	0.68	88.0	0.74
3 (nov) vs. Ref	86.0	0.67	83.0	0.65	82.0	0.57	77.5	0.56
all	84.6 (81.1; 88.0)	0.69 (0.62; 0.76)	81.8 (78.1; 85.6)	0.64 (0.56; 0.71)	86.3 (82.9; 89.6)	0.73 (0.66; 0.79)	82.7 (79.0; 86.3)	0.65 (0.58; 0.73)

Interrater agreement (Brennan and Prediger kappa statistic) between the three independent reviewers and the reference (Ref), as well as between all individual reviewers (all). *Κ*, kappa statistic; 95% CI, confidence interval; exp, experienced MBW operator; nov, novice MBW operator.

### 3.4 Comparison of MBW outcomes

In both setups LCI was higher in the CF group compared to the healthy children (Supplementary Table S3). Systematic visual quality control had no substantial influence on test results. There was no significant difference in LCI and FRC of acceptable trials between the reviewers, neither per setup nor per disease group within the setups (Supplementary Table S3).

## 4 Discussion

In this study, we developed an easy-to-use computer program for systematic visual quality assessment of infant SF_6_-MBW measurements collected with two widely used setups (Spiroware and WBreath). The software supports a swift and systematic visual assessment with straightforward documentation of the decision to accept/reject, the occurrence of artefacts, and individual comments, resulting in a good agreement among experienced and even novice users.

Artefacts such as leaks or sighs are recurrent in infant MBW measurements and can influence both the magnitude and variability of outcomes (1, 23). However, given the challenges of obtaining infant MBW measurements (especially during natural sleep) and a trend to obtain regular measurements in CF patients increasingly earlier (24, 25), there is a need for a quality control approach that rejects as few measurements as possible while providing robust results. Infant SF_6_-MBW measurements, especially those gathered using the Exhalyzer D/WBreath setup, rely on correct software settings and expose the operator to a multitude of signal processing steps as well as the (visual) quality control assessment (5, 11, 14, 26, 27). Therefore, until a comprehensive software solution (with options for data acquisition, signal processing, quality control, and reporting) becomes available from manufacturers, users have to rely on support from the scientific community. In this study, our tool enabled a user inexperienced with the MBW method to perform a visual quality control of infant SF_6_-MBW measurements comparable to that performed by experienced reviewers. Although the novice reviewer was more cautious and rejected more washouts, there was no substantial difference in the main MBW outcomes FRC and LCI. Additionally, an experienced user was able to significantly reduce the time required to perform and systematically document visual quality control for both infant setups, WBreath and Spiroware. When assessing WBreath-MBW data with the newly developed tool, the reference user performed five times faster compared to the standard analysis software.

The general criteria of our visual assessment were identical to the current ATS/ERS consensus statement (1), but our definition of the critical phase of washout differed. Instead of the 10 breaths before reaching equilibration (in the washin) and the first 10 breaths of the washout (1), we extended the critical phase to five breaths before washout start to five breaths after reaching the test-end criterion (1/40th of the starting tracer gas-concentration) and provide an automated phase identification for the washin and the washout as visual aid to the user. With the inclusion of the test-end criterion into the critical phase, we intend to ensure that artefacts at the end of the washout (even after the first ten breaths of the washout start) are included in the quality control assessment. This is of particular importance because small changes in the end-tidal tracer gas concentration (possibly caused by artefacts) during this phase hold potential to significantly influence the end-of-test criterion and thus the main outcome LCI (16).

We derived the artefact categories provided in our tool (leak, sigh, irregular breathing, and breath hold) from the current ATS/ERS consensus statement (1), and added quantifiable thresholds wherever possible. For example, we defined a sigh as a marked increase in tidal volume of at least 1.5-fold of the median tidal volume during the critical periods (23) and required breath holds to affect the flow as well as additional signals for a duration of at least two regular tidal breaths (28). Users of our tool are supported with an automated sigh detection as well as an option for heat map visualization of tidal volume per breath.

We recently identified and characterized significant measurement and signal correction errors in two main MBW devices, (i) a sensor-crosstalk error in the Exhalyzer D device (Eco Medics AG, Switzerland) (16), and (ii) errors in the respiratory exchange ratio-based adjustment of the measured CO_2_ concentration as well as a dependence on ambient humidity in the molar mass-sensor in the EasyOne Pro LAB device (ndd Medizintechnik AG, Switzerland) (29). In both devices, the errors lead to an overestimation of the resulting tracer gas concentration (16, 30). While software updates and changes in data analysis have happened before (5), their impact has never been of such a magnitude (31). In times of recurring software updates, a systematic review and quality control of existing MBW datasets is essential. Our software supports an approach for better standardization of infant SF_6_-MBW quality control and main outcomes between examiners and can thus assist users investigating large and/or longitudinal infant MBW datasets.

### 4.1 Strengths and limitations

The newly developed tool provides a framework for swift and systematic visual assessment of SF_6_-MBW measurements in infants, with straightforward documentation and good agreement among experienced and novice users. While the tool can read Spiroware raw data (A-files) and perform all underlying signal processing steps, we were not able to read original WBreath raw files (.brw files) in all cases. Therefore, a signal analysis in the WBreath software including a data export of corrected signals remains necessary before data visualization with our tool is possible. Our reference dataset consisted of one trial per measurement occasion, thus hampering the evaluation of intra-test variability. However, the newly developed tool will allow a systematic approach with a simple and user-friendly application interface.

## 5 Conclusion

The visual quality control tool supports the systematic retrospective assessment of predefined visual quality control criteria of infant SF_6_-MBW measurements. The tool proved to be applicable by three independent reviewers. Systematic visual quality control had no substantial influence on MBW outcomes. Therefore, the visual quality control tool can be applied reliably even by inexperienced users with comparable MBW results.

## Data Availability

The data that support the findings of this study are available from the corresponding author upon reasonable request.
