# Supplementary material for: A computerized tool for the systematic visual quality assessment of infant multiple-breath washout measurements
Source: Front Pediatr. 2024 Jun 6;12:1393291. doi: 10.3389/fped.2024.1393291 (PMC11191423; doi:10.3389/fped.2024.1393291)
Supplement: Supplementary file 1 [file Datasheet1.pdf]

A computerized tool for the systematic visual quality assessment of infant multiple-breath washout measurements

- Supplementary Material -

Marc-Alexander Oestreich[1] (https://orcid.org/0000-0001-9641-3691)

Isabelle Doswald[1]

Yasmin Salem[1] (https://orcid.org/0000-0001-8731-0885)

Noemi Künstle[2]

Florian Wyler[1] (https://orcid.org/0000-0002-1232-1392)

Bettina S. Frauchiger[1] (https://orcid.org/0000-0002-9519-9328)

Anne-Christianne Kentgens[1]

Philipp Latzin[1] (https://orcid.org/0000-0002-5239-1571)

Sophie Yammine[1] (https://orcid.org/0000-0001-7720-3445).

[1]Division of Paediatric Respiratory Medicine and Allergology, Department of Paediatrics, Inselspital, Bern University Hospital, University of Bern, Switzerland.

[2]University of Basel, Children's Hospital (UKBB), Basel 4056, Switzerland.

Corresponding author:

Sophie Yammine, MD, PhD

Inselspital

Bern University Children’s Hospital, Julie-von-Jenner-Haus

Freiburgstrasse 15, CH-3010 Bern, Switzerland

Email: sophie.yammine@insel.ch

Artefacts in multiple-breath washout measurements

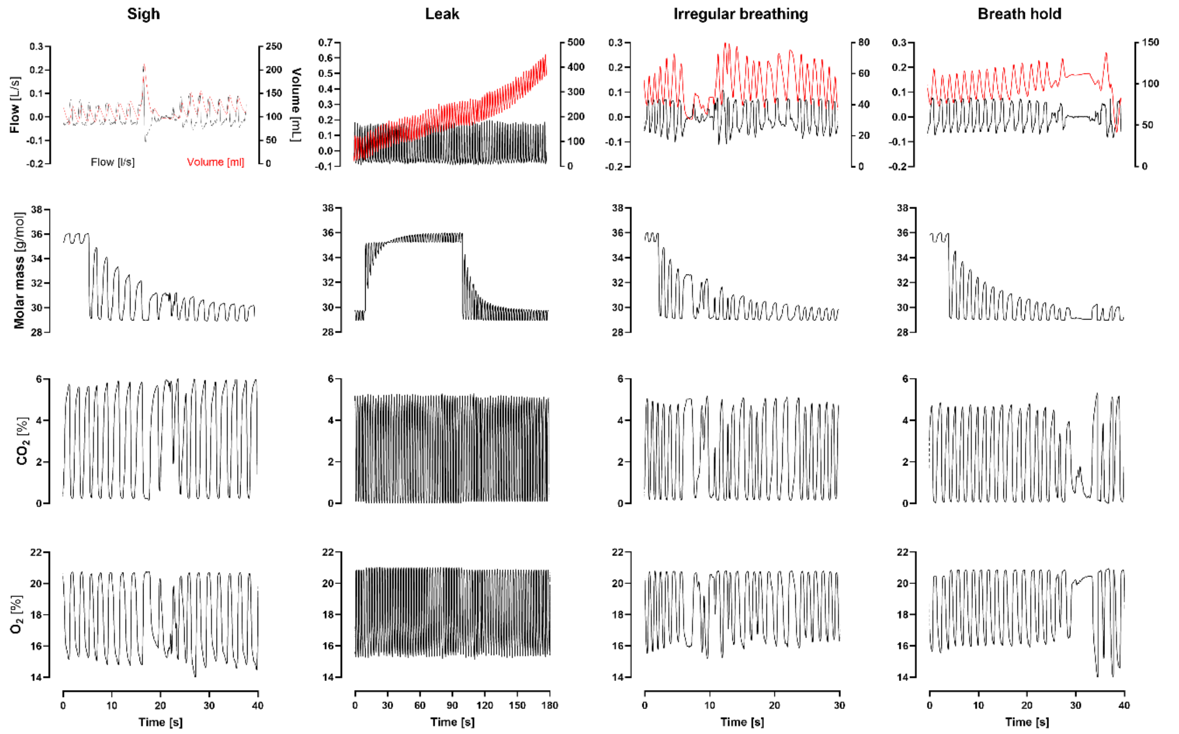

Supplementary figure 1. Artefacts in multiple-breath washout measurements. Shown are signal traces of flow [L/s] and volume [mL], molar mass [g/mol], carbon dioxide [%], and oxygen [%] from infant SF_6-MBW measurements gathered with the Exhalyzer D/Spiroware (Eco Medics AG) MBW setup. The columns depict four examples for possible artefacts (sigh, leak, irregular breathing pattern, and breath hold).

Study population characteristics

**A)**	**Spiroware setup** (n=200)		**WBreath setup** (n=200)	
	Healthy	CF	Healthy	CF
**Raw data files** (n, %total)	84 (42%)	116 (58%)	148 (74%)	52 (26%)
**Subjects** (n, %female)	51 (51%)	69 (52%)	120 (39%)	45 (42%)
**Age**, weeks (median, range)	6.0 (3.3; 72.3)	13.1 (3.7; 69.4)	5.2 (3.6; 58.7)	13.9 (4.0; 82.0)

Supplementary table 1. Study population characteristics. Shown are absolute and relative numbers of raw data files and study participants per setup (Spiroware and WBreath), as well as median (range) age in weeks. Abbreviations: Healthy: healthy controls; CF: cystic fibrosis.

Time comparison

Time required [s]	**Spiroware setup** (n=5)					**WBreath setup** (n=5)				
	**Spiroware 3.3.1**	**visual QC tool**	**mean difference**	**95% CI**	**p-value**	**WBreath 3.52.3**	**visual QC tool**	**mean difference**	**95% CI**	**p-value**
to boot and import raw data	531.0	55.5	475.5	-	-	9.3	59.2	49.9	-	-
for QC and documentation	16.5	11.4	5.1	0.7 to 9.4	**0.031**	43.5	8.6	34.9	28.5 to 41.3	**0.0001**

Supplementary table 2. Time comparison between standard analysis software and the developed visual QC tool. Time required to boot the analysis software and import a dataset of five raw data files (A-Files for Spiroware; BRW-Files for WBreath; ASCII export of BRW-Files for the WBreath visual QC tool) and to perform a visual quality control including the documentation of a) the decision to accept/reject, b) identified visual artefacts, and c) reason for exclusion. Abbreviations: s: seconds; QC: visual qualitiy control; CI: confidence interval.

Impact on MBW outcomes

	**Spiroware setup** (n=200)				**WBreath setup** (n=200)			
	**REF**	**1**	**2**	**3**	**REF**	**1**	**2**	**3**
Acceptable wash-outs (n)	126	131	141	108	123	113	118	99
FRC (ml)	117.3 (54.1)	118.0 (52.1)	117.5 (53.0)	109.3 (46.1)	123.3 (58.4)	121.6 (61.5)	129.6 (66.3)	122.4 (64.2)
LCI (TO)	8.0 (1.5)	8.0 (1.4)	8.0 (1.5)	8.0 (1.6)	7.2 (2.0)	7.5 (1.6)	7.2 (2.6)	7.6 (1.5)
	Healthy				*Healthy*			
Acceptable wash-outs (n)	56	58	58	52	84	83	79	71
FRC (ml)	85.7 (27.8)	87.2 (27.0)	85.5 (27.6)	82.8 (21.7)	99.0 (14.2)	99.3 (18.2)	101.2 (23.2)	99.0 (12.0)
LCI (TO)	8.0 (1.7)	8.0 (1.6)	8.0 (1.6)	8.1 (1.9)	7.1 (1.1)	7.3 (1.0)	7.0 (1.7)	7.2 (0.8)
	CF				*CF*			
Acceptable wash-outs (n)	70	73	83	56	39	30	39	28
FRC (ml)	142.6 (56.7)	142.5 (54.3)	139.9 (55.2)	133.9 (49.1)	175.7 (79.9)	183.1 (91.2)	187.2 (85.8)	181.7 (97.5)
LCI (TO)	8.1 (1.4)	8.0 (1.3)	8.1 (1.5)	7.9 (1.3)	7.3 (3.2)	8.0 (2.7)	7.5 (3.8)	8.7 (2.2)

Supplementary table 3. Impact of the decision to accept/reject the washout on MBW outcomes. Mean MBW outcomes displayed as mean (SD). Abbreviations: REF: reference; 1-3 individual reviewers; FRC: functional residual capacity; LCI: lung clearance index [turnover]; Healthy: healthy controls; CF: cystic fibrosis.

How to set up the visual QC tool

We developed a software package for the visualization of (raw Spiroware and signal processed WBreath) infant SF_6-MBW signal traces to support a systematic assessment of pre-defined visual QC criteria. The tool is available to researchers as a compiled software package for the analysis of WBreath or Spiroware infant SF_6-MBW data.

1. Please find the compiled software packages here: https://doi.org/10.6084/m9.figshare.22193737.v2
2. To start the tool, please unpack the respective directory locally on your computer and double-click the .exe-file in the *dist**Spiroware**_infant_MBW_visual_QC* or *dist**WBreath**_infant_MBW_visual_QC* folder.
3. Upon launch, the tool guides the user through a number of selections for
   i. a configuration file (please find examples which can also be adapted (e.g., examiner identification) here: https://doi.org/10.6084/m9.figshare.22193737.v2),
   ii. an output directory on your local computer for the results (spreadsheet), and
   iii. raw spiroware (A-files) or signal processed WBreath (text) files for quality assessment.
4. Upon selection of the data, the user interface will appear and visualize the first measurement (supplemental figure 2). The user can assess all of the selected measurements or close the tool and save the results to the selected output directory.

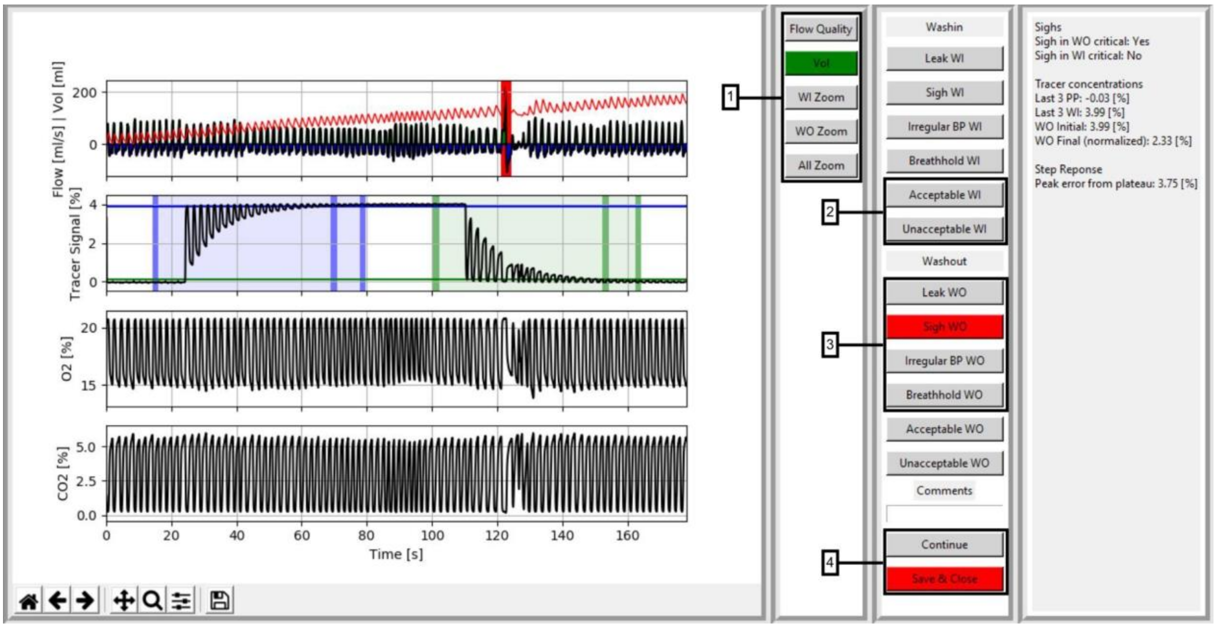

Supplementary figure 2. **User interface of the visual quality control tool for infant SF_6-MBW measurements.** The visual quality control tool provides the user with a visualization in four panels. First, a visualization of the signal traces for flow [ml/s] and volume [ml], a computed tracer gas concentration [%], oxygen [%], and carbon dioxide [%] concentrations. The critical periods are highlighted by blue and green areas in the tracer signal, with vertical bars indicating i) the beginning of the critical phase, ii) the 2.5% criterion, and iii) the end of the critical phase. In this example, a sigh (= a breath with a significant increase in tidal volume above 1.5 times the median tidal volume) during the washout is highlighted in red by the automatic sigh detection and the user is provided with a comment (panel four). The second panel provides the user with display options (e.g., zoom for the washin and washout-phase and a heat map of tidal volume per breath (flow quality; box 1). The third panel provides options for systematic assessment (e.g., to accept/reject (box 2), to identify and document artefacts (leak, sigh, irregular breathing pattern, breath hold; box 3), and allows for individual comments. Additionally, the user can continue to the next measurement or save and close the visual quality assessment (box 4). The fourth panel provides additional information to the operator (e.g., automatic identification of sighs, deviations in step response-correction, or molar mass steps between phases).

Summary on applied visual quality control criteria

Based on available recommendations for SF_6-MBW tests in sleeping infants, we aimed to develop a protocol and a computerized tool to systematically evaluate the numerical (e.g., end-of test criterion, stable tracer-gas equilibrium at the end of the washin) and visual quality of the washin and washout phases of SF_6-MBW measurements. While numerical quality criteria (e.g., reaching the 2.5% criterion or achieving two or more technically acceptable measurements per visit) are relatively easy to define and verify, visual quality control criteria (e.g., leak, sigh, irregular breathing pattern, and breathhold; supplemental figure 1) can pose a challenge to the user (supplemental figure 3).

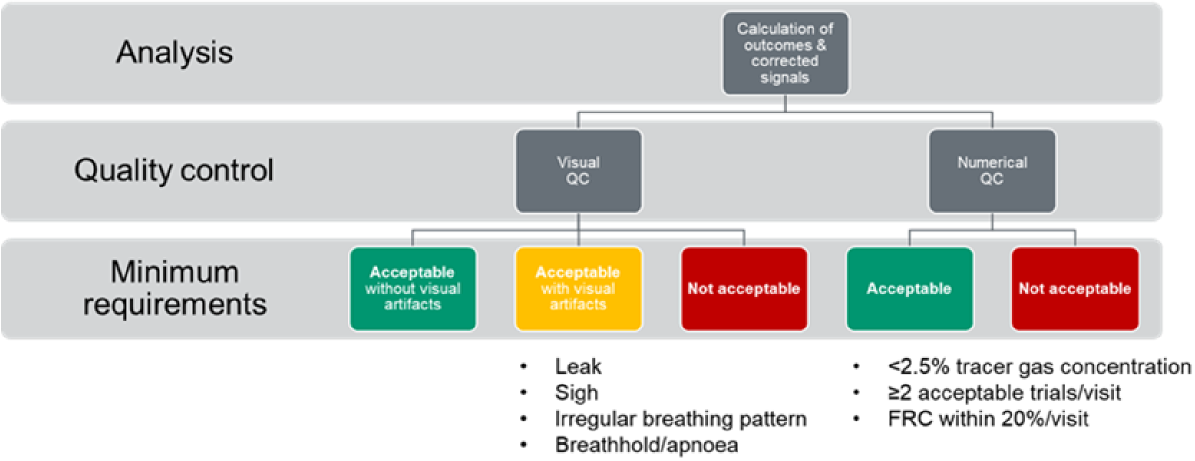

Supplementary figure 3. Quality control protocol for infant SF_6-MBW measurements. Based on a bilateral approach of numerical and visual QC, we propose to first perform signal processing and outcome calculation (numerical QC) followed by a visual assessment of corrected signals (visual QC). Abbreviations: QC quality control; FRC functional residual capacity.

We propose the following steps when performing a visual quality assessment of infant SF_6-MBW measurements with our tool:

1. Inspect the signal quality and overall course of signals (use the “flow quality” to apply a heat map visualisation of all tidal breaths).
2. Inspect the critical phases (use the zoom option for washin and washout).

3. Document incomplete measurements using an individual comment. We propose the following format:
    a. Complete pre-phase and washin but (visually) incomplete washout: **incomplete WO** (washout)
    b. Complete pre-phase but (visually) incomplete washin: **incomplete WI** (washin)
    c. Incomplete prephase: **incomplete**
    d. Erroneous signals: **Error**
4. We propose to identify and document visual artefacts as defined in Table 2 in the manuscript. We describe general quality criteria based on current ATS/ERS recommendatins and propose four common artefacts (leak, sigh, irregular breathing, and breathhold). Any measurements that are conspicuous should be described with an individual comment and, if necessary, identified as unacceptable.
5. Finally, computed outcomes, and both the numerical and the visual quality control should be merged for each individual measurement.
